# Supplementary material for: PeptiVerse: A Unified Platform for Therapeutic Peptide Property Prediction
Source: bioRxiv. 2026 Jan 3:2025.12.31.697180. Preprint. [Version 1] doi: 10.64898/2025.12.31.697180 (PMC12773018; doi:10.64898/2025.12.31.697180)
Supplement: Supplement 1 [file NIHPP2025.12.31.697180v1-supplement-1.pdf]

bioRxiv preprint doi: https://doi.org/10.64898/2025.12.31.697180; this version posted January 3, 2026. The copyright holder for this preprint (which was not certified by peer review) is the author/funder, who has granted bioRxiv a license to display the preprint in perpetuity. It is made available under aCC-BY 4.0 International license.

Supplementary Information

Supplementary Methods

Half-Life Data Preparation Peptide half-life can be strongly influenced by terminal modifications, D-amino acids, unnatural residues, and backbone cyclization [Mathur et al., 2016]. To preserve chemically relevant information, peptides were represented using SMILES whenever available, while sequence-based representations were also retained. For datasets lacking canonical SMILES, structures were generated from sequence-level information using the `fasta2smi` utility from `p2smi` [Feller and Wilke, 2025b]. Tokenized sequences with lengths over 200 were filtered out to avoid outliers.

Half-life entries from PepTherDia [D'Aloisio et al., 2021] were provided as canonical SMILES with qualitative or ranged lifespan descriptions. PepTherDia contains mostly either entries with unnatural amino acids or chemical modification at the ends, or sequences with cyclic connections. Entries such as "cannot be calculated" were excluded. Ranges such as "1–6 min" were approximated using the midpoint (taken as 900 seconds), and qualitative descriptions like "10 minutes or less" were converted to fixed values (600 seconds). THPdb2 [Jain et al., 2024] also reported half-life values using descriptive or numerical formats, but does not provide canonical SMILES. In addition, peptide sequences may omit D-amino acid annotations or represent noncanonical residues using placeholder symbols ("X"). To resolve these ambiguities, SMILES representations were retrieved based on therapeutic names when available and cross-validated against reported chemical formulas using PubChem [Kim et al., 2023].

Clear numerical entries were standardized by converting all measurements to seconds, for example, "0.43 hours" to 1548 seconds, and "3.8 ± 0.6 hrs" to 13,680 seconds using the central value. When multiple half-life measurements were reported for a single peptide under different experimental conditions, all entries were retained to reflect condition-dependent variability. The heterogeneity of half-life annotations reflects variability in experimental protocols rather than preprocessing artifacts. For PEPlife [Mathur et al., 2016], canonical SMILES and unambiguous compound identifiers were generally unavailable, and annotations frequently mixed D-amino acids and unnatural residues based on literature reports. Due to inconsistent or incomplete chemical specifications, explicit reconstruction of most unnatural amino acids was not attempted. Instead, peptides containing N–C cyclization or D-amino acids were treated as modified entries, while only non-cyclic, non-`X`-containing sequences with standard L-amino acid chirality were considered wild-type. D-amino-acid-containing SMILES were generated using the SMILES2PEPTIDE transformer from Tang et al. [2025c].

After cleaning, approximate SMILES construction, unit normalization, and duplicate removal based on exact matches of sequence and half-life duration, the final dataset contained 245 unique peptide entries, including both wild-type and chemically modified peptides. Among these, 130 entries correspond to amino acid–based peptide representations without explicit noncanonical modifications. For interpretability, all half-life values were converted from seconds to hours for reporting and evaluation.

bioRxiv preprint doi: https://doi.org/10.64898/2025.12.31.697180; this version posted January 3, 2026. The copyright holder for this preprint (which was not certified by peer review) is the author/funder, who has granted bioRxiv a license to display the preprint in perpetuity. It is made available under aCC-BY 4.0 International license.

Supplementary Figures and Tables

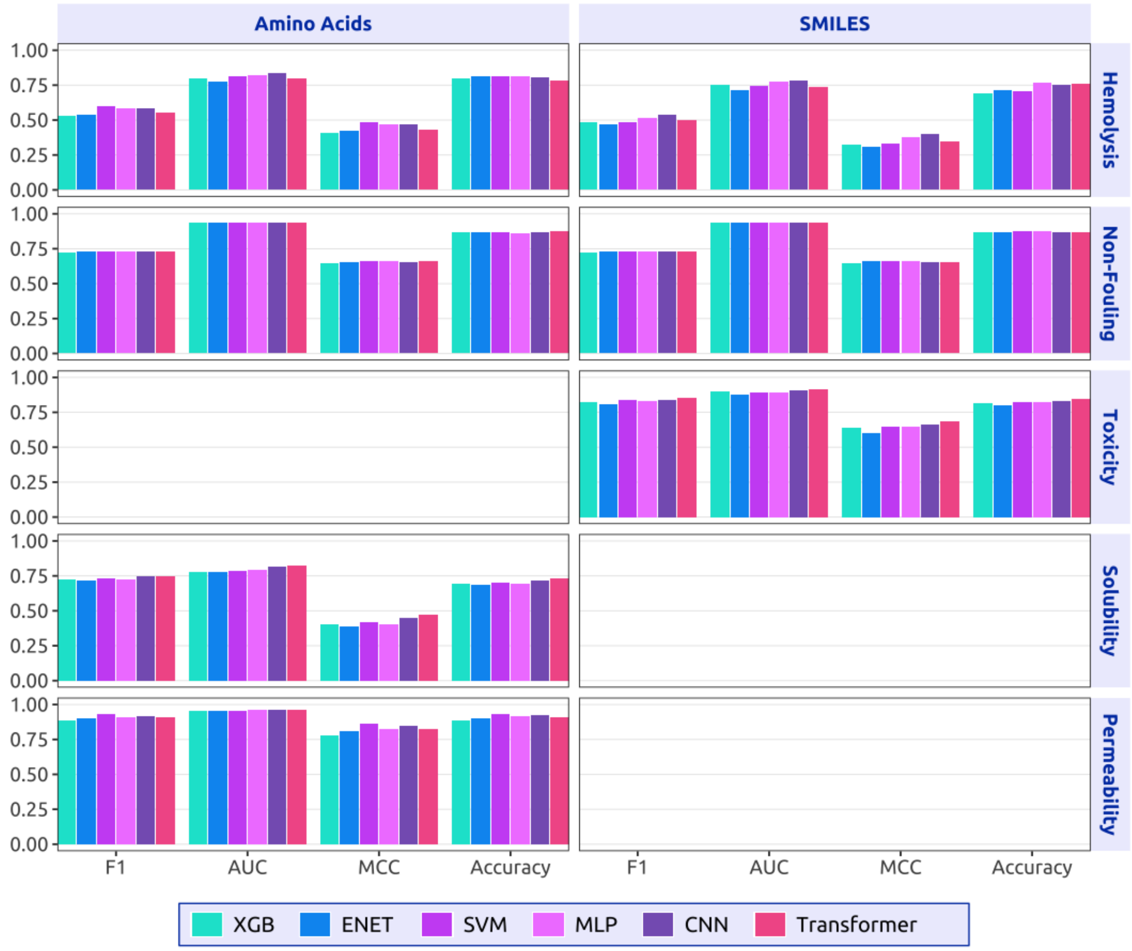

Figure S1: **Per class performance across models.** The best performing models were selected based on validation performance, refit on the validation set, and evaluated for final reporting. Properties without data are not reported.

bioRxiv preprint doi: https://doi.org/10.64898/2025.12.31.697180; this version posted January 3, 2026. The copyright holder for this preprint (which was not certified by peer review) is the author/funder, who has granted bioRxiv a license to display the preprint in perpetuity. It is made available under aCC-BY 4.0 International license.

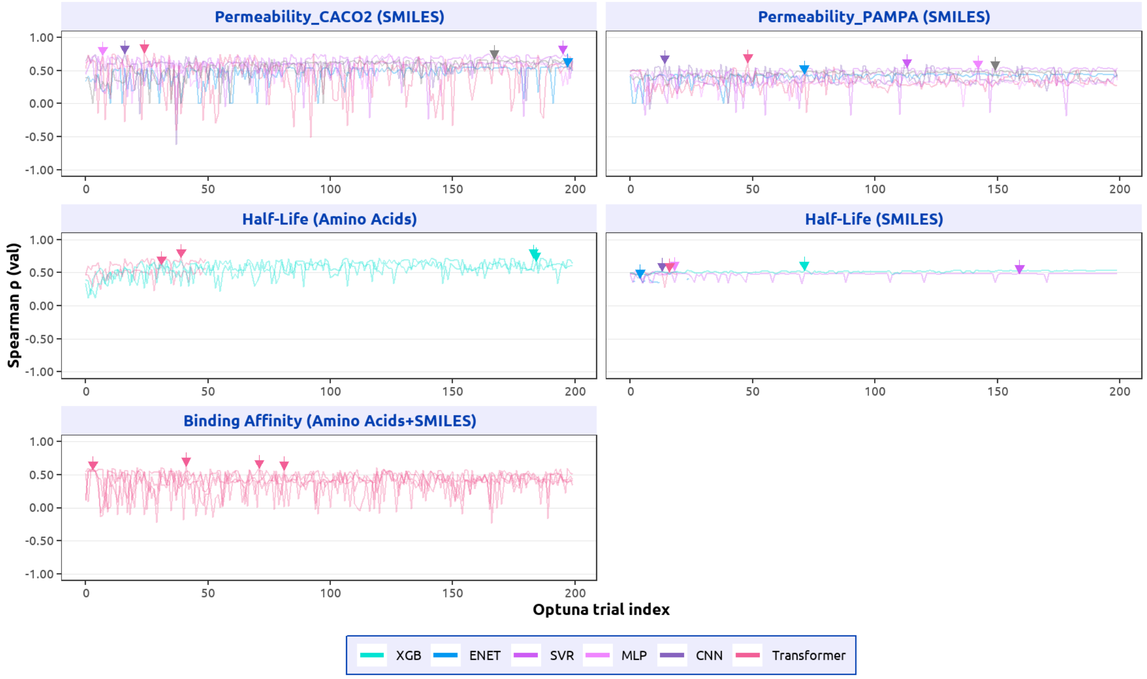

Figure S2: **Optuna optimization traces for regression models.** The arrow marks the selected trial with the best Spearman correlation (ρ) for each run. Machine-learning-based algorithms (XGB, ENET, SVR) were optimized for 200 trials, and neural-network-based algorithms (MLP, CNN, Transformers, where applicable) were optimized for 50 trials. SMILES tokenization results in longer input sequences and, therefore, slower training for neural-network-based models. The model choices for half-life and binding affinity reflect computational constraints and design considerations.

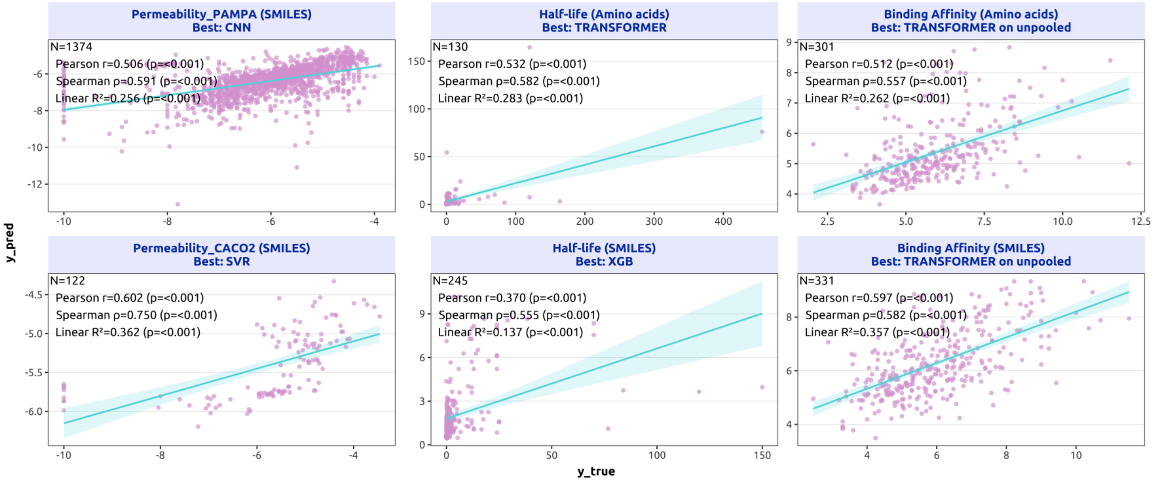

Figure S3: **Top model performance for regression models.** The best-performing models were selected and refit on the validation set for final evaluation. For half-life prediction, models were trained using cross-validation. The correlations are reported based on out-of-fold predictions collected during training. Specifically, in k-fold cross-validation, each fold's predictions were generated by models trained on the remaining $k-1$ folds, and the final results aggregate all held-out predictions.

bioRxiv preprint doi: https://doi.org/10.64898/2025.12.31.697180; this version posted January 3, 2026. The copyright holder for this preprint (which was not certified by peer review) is the author/funder, who has granted bioRxiv a license to display the preprint in perpetuity. It is made available under aCC-BY 4.0 International license.

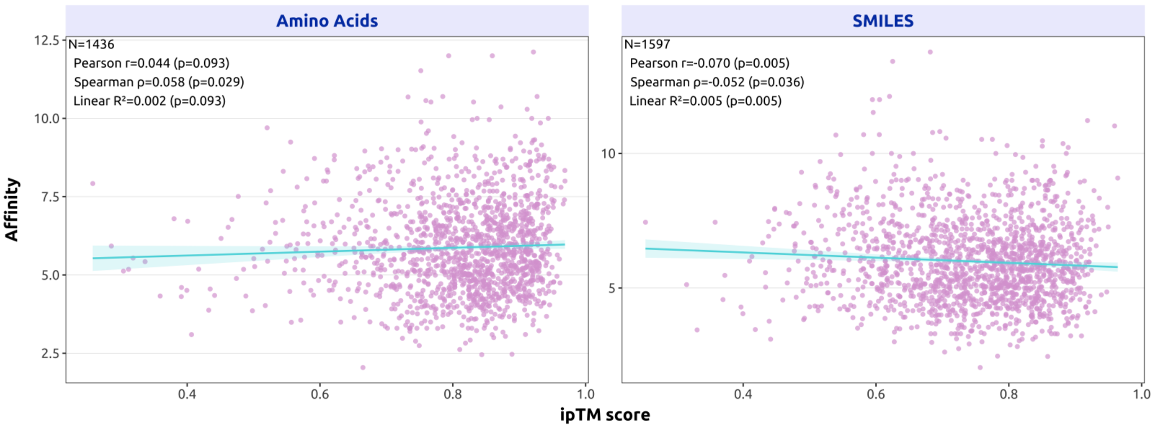

Figure S4: **Correlation between normalized experimental affinity score and Openfold3 ipTM score.**

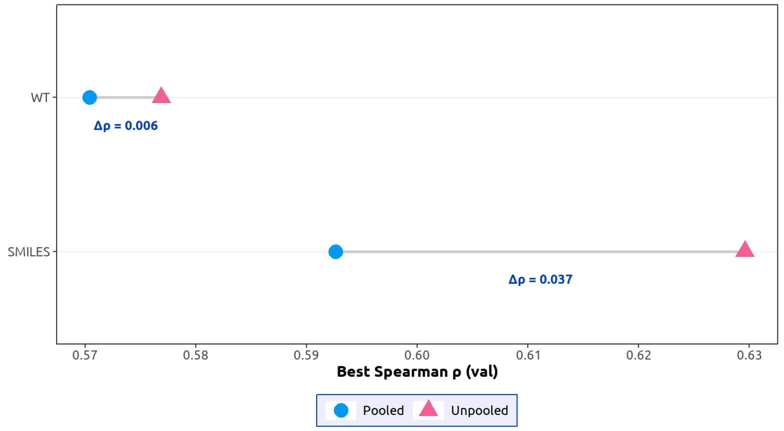

Figure S5: **Unpooled versus pooled embeddings for binding affinity.** Best validation Spearman ρ from Optuna optimization for WT and SMILES inputs are shown. The unpooled embeddings outperform pooled embeddings in both settings (ρ difference shown).

bioRxiv preprint doi: https://doi.org/10.64898/2025.12.31.697180; this version posted January 3, 2026. The copyright holder for this preprint (which was not certified by peer review) is the author/funder, who has granted bioRxiv a license to display the preprint in perpetuity. It is made available under aCC-BY 4.0 International license.

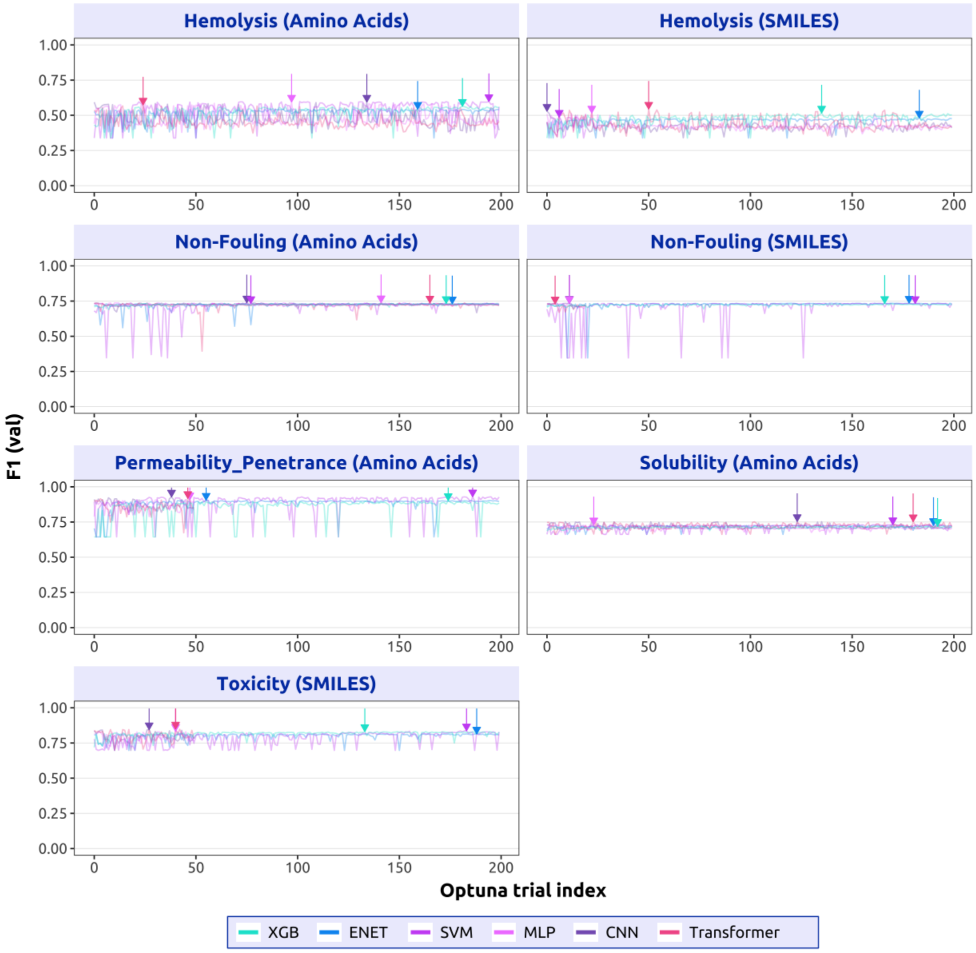

Figure S6: **Optuna optimization traces.** The arrow marks the selected trial with best F1 value for each run. All models were optimized for 200 trials.

bioRxiv preprint doi: https://doi.org/10.64898/2025.12.31.697180; this version posted January 3, 2026. The copyright holder for this preprint (which was not certified by peer review) is the author/funder, who has granted bioRxiv a license to display the preprint in perpetuity. It is made available under aCC-BY 4.0 International license.

Table S1: **Final classification thresholds.**

Properties	Best Model		Classification Thresholds		Best F1	
	Amino acid	SMILES	Amino acid	SMILES	Amino acid	SMILES
Hemolysis	SVM	Transformer	0.2521	0.4343	0.5975	0.5211
Non-Fouling	MLP	ENET	0.57	0.6969	0.7388	7356
Solubility	CNN	-	0.377	-	0.7537	-
Permeability (Penetrance)	SVM	-	0.5493	-	0.9291	-
Toxicity	-	Transformer	-	0.3401	-	0.8455

Table S2: **XGBoost hyperparameters for classification and regression tasks.** The ranges indicate Optuna search space boundaries. †Indicates log-uniform sampling in log space.

XGBoost Hyperparameters		
**Hyperparameter**	**Classification**	**Regression**
Objective	`binary:logistic`	`reg:squarederror`
Eval metric	`logloss`	`rmse`
Lambda (λ)	$[10^{-8}, 50]^\dagger$	$[10^{-10}, 10^2]^\dagger$
Alpha (α)	$[10^{-8}, 50]^\dagger$	$[10^{-10}, 10^2]^\dagger$
Gamma (γ)	$[0, 10]$	$[0, 10]$
Max depth	$[2, 15]$	$[2, 16]$
Min child weight	$[1, 500]$	$[10^{-3}, 500]^\dagger$
Subsample	$[0.5, 1.0]$	$[0.5, 1.0]$
Colsample by tree	$[0.3, 1.0]$	$[0.3, 1.0]$
Learning rate	$[10^{-3}, 0.3]^\dagger$	$[10^{-3}, 0.3]^\dagger$
Boosting rounds	$[50, 1500]$	$[50, 2000]$
Early stopping	$[20, 200]$	$[20, 200]$
Tree method	`hist`	`hist`

Table S3: **Elastic Net hyperparameters for classification and regression tasks.** †Indicates log-uniform sampling. The class weight and selection are categorical choices.

Elastic Net Hyperparameters		
**Hyperparameter**	**Classification**	**Regression**
Model type	`cuLogReg`, penalty="elasticnet"	`cuElasticNet`
Solver	`qn` (cuML)	cuML
C (inverse regularization)	$[10^{-4}, 10^3]^\dagger$	-
α (regularization)	-	$[10^{-8}, 10]^\dagger$
ℓ_1 ratio	$[0, 1]$	$[0, 1]$
Class weight	{`balanced`}	-
Selection	-	{`cyclic`, `random`}
Max iterations	$[200, 5000]$	$[1000, 20000]$
Tolerance	$[10^{-6}, 10^{-2}]^\dagger$	$[10^{-6}, 10^{-2}]^\dagger$

bioRxiv preprint doi: https://doi.org/10.64898/2025.12.31.697180; this version posted January 3, 2026. The copyright holder for this preprint (which was not certified by peer review) is the author/funder, who has granted bioRxiv a license to display the preprint in perpetuity. It is made available under aCC-BY 4.0 International license.

Table S4: **Support Vector Machine hyperparameters for classification and regression tasks.** †Indicates log-uniform sampling. γ only applies to non-linear kernels.

Support Vector Machine Hyperparameters		
**Hyperparameter**	**Classification**	**Regression**
Model type	`cuSVC (cuML)`	`SVR (scikit-learn)`
Kernel	{rbf, linear, poly, sigmoid}	{rbf, linear, poly, sigmoid}
C (regularization)	$[10^{-3}, 10^{3}]^{\dagger}$	$[10^{-3}, 10^{3}]^{\dagger}$
γ (kernel coefficient)	$[10^{-6}, 10]^{\dagger}$ or `scale`	$[10^{-6}, 10]^{\dagger}$ or `scale`
ϵ (tube width)	-	$[10^{-4}, 1]^{\dagger}$
Class weight	{`balanced`}	-
Max iterations	$[200, 5000]$	-
Tolerance	$[10^{-6}, 10^{-2}]^{\dagger}$	-

Table S5: **Neural network hyperparameters for classification and regression tasks.** All models share the same base hyperparameters, with additional hyperparameters introduced only where required by the specific architecture. †Indicats log-uniform sampling. ‡The Huber δ controls outlier sensitivity.

Neural Network Hyperparameters		
**Hyperparameter**	**Classification**	**Regression**
**Shared parameters (all architectures)**		
Learning rate	$[10^{-5}, 3 \times 10^{-3}]^{\dagger}$	$[10^{-5}, 3 \times 10^{-3}]^{\dagger}$
Weight decay	$[10^{-8}, 10^{-2}]^{\dagger}$	$[10^{-10}, 10^{-2}]^{\dagger}$
Dropout rate	$[0, 0.5]$	$[0, 0.5]$
Batch size	{16, 32, 64}	{16, 32, 64}
Loss function	Cross-entropy	{MSE, Huber}
Huber $\delta^{\ddagger}$	-	$[0.5, 5]^{\dagger}$
**MLP-specific**		
Hidden units	{256, 512, 1024, 2048}	{256, 512, 1024, 2048}
**CNN-specific**		
Channels	{128, 256, 512}	{128, 256, 512}
Kernel size	{3, 5, 7}	{3, 5, 7}
Number of layers	$[1, 4]$	$[1, 4]$
**Transformer-specific**		
Model dimension (d_{model})	{128, 256, 384}	{128, 256, 384}
Attention heads	{4, 8}	{4, 8}
Number of layers	$[1, 4]$	$[1, 4]$
FFN dimension	{256, 512, 1024, 1536}	{256, 512, 1024, 1536}

bioRxiv preprint doi: https://doi.org/10.64898/2025.12.31.697180; this version posted January 3, 2026. The copyright holder for this preprint (which was not certified by peer review) is the author/funder, who has granted bioRxiv a license to display the preprint in perpetuity. It is made available under aCC-BY 4.0 International license.

Table S6: **Binding affinity model hyperparameters.** A cross-attention architecture was used for peptide-protein binding prediction. †Indicates log-uniform sampling.

**Binding Affinity Predictor Hyperparameters**	
**Hyperparameter**	**Value / Range**
**Optimizer settings**	
Learning rate (AdamW)	$[10^{-5}, 3 \times 10^{-3}]^{\dagger}$
Weight decay	$[10^{-10}, 10^{-2}]^{\dagger}$
**Architecture parameters**	
Hidden dimension	{256, 384, 512, 768}
Attention heads	{4, 8}
Cross-attention layers	$[1, 4]$
Dropout rate	$[0, 0.4]$
**Training configuration**	
Batch size	{16, 32, 64, 128}
Auxiliary loss weight (λ_{cls})	$[0.1, 2.0]^{\dagger}$
Primary loss	MSE (binding affinity)
Auxiliary loss	BCE (binding classification)
Max epochs	50
Early stopping patience	10
